# Protective effects of *Zingiber officinale* Roscoe in obstetric antiphospholipid syndrome based on systems pharmacology and molecular docking

**DOI:** 10.1097/MD.0000000000048706

**Published:** 2026-05-08

**Authors:** Bo Du, Hongmei Wang, Qun Wang, Ning He, Kai Wang, Hui Li

**Affiliations:** a Department of Gynecology and Obstetrics, People’s Hospital Affiliated to Shandong First Medical University, Jinan, Shandong Province, China.

**Keywords:** anticoagulation, immunoregulation, molecular docking, obstetric antiphospholipid syndrome, systems pharmacology

## Abstract

Antiphospholipid syndrome is an autoimmune disorder of unknown etiology. The anti-inflammatory and immunomodulatory properties of *Zingiber officinale* Roscoe may provide a novel approach for potentially preventing obstetric antiphospholipid syndrome (OAPS). Based on several public databases, compounds and potential drug targets of *Z officinale* Roscoe were screened, and a compound-target network was constructed using Cytoscape. The targets of OAPS were identified from GeneCards. The common genes of the disease targets and compound targets were defined as hub genes. Protein–protein interaction, Gene Ontology, and Kyoto Encyclopedia of Genes and Genomes analyses were performed, and the OAPS pathway was obtained. Molecular docking was used to verify the binding ability of the effective compounds and targets. In total, 298 compounds were identified as components of *Z officinale* Roscoe. Among these, 12 active compounds exhibited an oral bioavailability of ≥30% and drug-likeness of ≥0.14, with 99 associated targets. A total of 28 common targets were identified as hub genes. In addition, the beneficial effects of *Z officinale* Roscoe in OAPS management are thought to be mediated by anticoagulant, anti-inflammatory, and immunomodulatory mechanisms. These processes are regulated by 9 biological signaling pathways involved in the protection against OAPS. Molecular docking showed that the binding affinities of tumor protein 53 with beta-sitosterol, tumor protein 53 with stigmasterol, prostaglandin G/H synthase 2 with beta-sitosterol, and prostaglandin G/H synthase 2 with stigmasterol were −6.651, −6.709, −7.099, and −6.829, all of which had high binding affinities. Overall, systematic pharmacology and molecular docking provide a new theoretical basis and research insight for the prevention of OAPS.

## 1. Introduction

Antiphospholipid syndrome (APS) is characterized by recurrent thrombosis or obstetric diseases. Antiphospholipid antibodies (APLAs) induce cell activation and inhibition of natural anticoagulant and fibrinolytic systems, and complement activation is one of the pathophysiological mechanisms in patients with APS.^[1]^ In obstetric antiphospholipid syndrome (OAPS), low-molecular-weight heparin, unfractionated heparin, or aspirin may increase the rate of continued pregnancy.^[2]^ However, close monitoring of prothrombin levels is required. Anti-inflammatory and immunomodulatory agents have also been proposed as potential new protective or treatment strategies for OAPS.^[1]^

*Zingiber officinale* Roscoe (ZOR) is a traditional food medicine similar to traditional Chinese medicine. Additionally, modern pharmacological research has demonstrated its therapeutic effects on the digestive, circulatory, respiratory, and central nervous systems, as well as its antioxidant,^[3,4]^ antiangiogenic,^[5]^ anti-inflammatory,^[6]^ and immunomodulatory properties.^[4]^ Furthermore, ZOR’s anti-inflammatory and immunomodulatory properties may offer a new approach for the protection of OAPS.

Recently, systems pharmacology applications have provided a basis for exploring the molecular mechanisms of action of the compounds used in traditional Chinese medicine. Molecular docking predicts the quantitative structure-activity relationships between compounds and their targets, enabling high-throughput screening of herbal monomers and offering new insights into drug discovery and design. No previous study has examined ZOR’s interaction with OAPS molecular targets through in silico methods. The aim was to employ this theory to identify putative molecular pathways and targets of ginger in OAPS and to explain the anticoagulant, anti-inflammatory, and immunomodulatory effects of ZOR as a potential agent for OAPS.

## 2. Materials and methods

### 2.1. Databases of active compounds

All phytochemicals identified in ZOR were manually retrieved from the Traditional Chinese Medicine Systems Pharmacology Database and Analysis Platform (https://www.tcmsp-e.com/#/home), the Shanghai Institute of Organic Chemistry of CAS Chemistry Database (DB/OL; https://organchem.csdb.cn/scdb/default.htm), and Traditional Chinese Medicine on Immuno-Oncology (http://tcmio.xielab.net/index).

### 2.2. Identifying potential active compounds

To identify the potential active compounds of ZOR, we used a method that combined oral bioavailability (OB), drug-likeness (DL), and Lipinski rules (LRs). In this study, the screening index for OB was set to ≥30% for this purpose.^[7]^ A DL value of ≥0.18 indicated that the compounds and drugs in the DrugBank database were similar. However, a literature review revealed that some known effective compounds have a DL value of 0.14. Therefore, in this study, a DL of ≥0.14 was used as the screening standard to identify all compounds with potential medicinal properties. The LRs^[8]^ screening criteria were as follows: molecular weight <500, number of hydrogen donors <5, number of hydrogen acceptors <10, and lipid–water distribution coefficient <5. Active oral drugs were considered to meet at least 3 of the aforementioned criteria.

### 2.3. Drug targets and disease targets

The targets of ZOR were obtained from the Traditional Chinese Medicine Systems Pharmacology Database and Analysis Platform and the Traditional Chinese Medicine on Immuno-Oncology databases and were mapped to the UniProt (http://www.uniprot.org/) database. We also built a compound-target (C-T) network using Cytoscape 3.10.3 (The Cytoscape Consortium, Seattle) to better understand the pathogenesis of this disorder and the pharmacological mechanism of action of ZOR. Targets for OAPS were retrieved using GeneCards: The Human Gene Database (https://www.genecards.org/), searching for “obstetric antiphospholipid syndrome and protein coding” as keywords on May 14, 2025. The 2 groups of targets intersected to screen for common targets. We overlapped the candidate targets of ZOR with OAPS therapeutic targets to obtain shared targets using the software: R (4.2.1; The R Foundation for Statistical Computing, Vienna, Austria), ggplot2 packages (Posit PBC, Boston), and the Venn Diagram package (University of California, Los Angeles [UCLA], Los Angeles) to visualize the results.

### 2.4. Construction of PPI and functional enrichment

To understand these shared target interactions, they were added to STRING (https://cn.string-db.org) to conduct network topology analysis. The core protein–protein interaction (PPI) network was analyzed using Cytoscape 3.10.3. Furthermore, we used the Cytoscape 3.10.3 Network Analyzer to examine the 2 most important topological parameters of network nodes: degree and betweenness. Gene Ontology (GO) and Kyoto Encyclopedia of Genes and Genomes (KEGG) analyses were performed to determine whether the selected targets were associated with OAPS. R software (4.2.1) was used to map potential targets by systematic annotation for the GO terms biological process (BP), molecular function (MF), cellular component (CC), and KEGG. The results were visualized using the ggplot2 package. We also built a target-pathway (T-P) network using Cytoscape 3.10.3.

### 2.5. OAPS pathway analysis

We constructed a complete “OAPS pathway” manually assembled by excluding, based on T-P network, pathways indirectly related or unrelated to OAPS based on the current trends in the clinical protection of OAPS and research on the pathogenesis of this disease.

### 2.6. Molecular docking verification

SwissDock (SIB Swiss Institute of Bioinformatics, Molecular Modeling Group, University of Lausanne, Lausanne, Switzerland, https://www.swissdock.ch) was used to analyze the binding affinities and modes of interaction between drug candidates and their targets. The AutoDock Vina (The Scripps Research Institute [Center for Computational Structural Biology, Forli Lab], La Jolla) scoring function incorporates 4 components: van der Waals forces, an undirected hydrogen-bond component, a hydrophobic factor, and a penalty for conformational entropy.^[9]^ Vina evaluates molecular interactions via trilinear interpolation of precomputed grid maps based on the target structure, and it employs the target structure for postprocessing minimization of docked poses. The docking box range was then selected based on the receptor’s active site. The molecular structures of the potentially active compounds were retrieved from PubChem (https://pubchem.ncbi.nlm.nih.gov/). The 3D coordinates of the targets were downloaded from the PDB (http://www.rcsb.org/pdb/home/home.do). Binding affinity reflects the degree of binding between drug molecules and proteins and is an important basis for screening potential drug molecules. If a drug molecule has high binding affinity for the target protein, it has greater potential to become an effective therapeutic drug.

## 3. Results

### 3.1. Screening active compounds

A total of 298 compounds derived from ZOR were identified by screening and deduplication of the 3 databases used. Twelve active compounds were obtained (Table 1), all of which had OB and DL values ≥30% and ≥0.14, respectively, which was consistent with those of the LRs. Therefore, we postulate that the above-mentioned ingredients may be key constituents in the treatment of OAPS using ZOR.

**Table 1:** The active compound and its parameters of ZOR.

ID	Molecule name	MW	Hdon	Hacc	OB (%)	DL
M1	6-shogaol	276.41	1	3	31.00	0.14
M5	EIC	280.5	1	2	41.90	0.14
M7	linolenic acid	278.48	1	2	45.01	0.15
M11	Diepicedrene-1-oxide	220.39	0	1	102.20	0.15
M2	6-gingerol	294.43	2	4	35.64	0.16
M10	Euxanthone	228.21	2	4	92.98	0.16
M8	Dihydrocapsaicin	307.48	2	4	47.07	0.19
M9	6-methylgingediacetate2	394.56	0	6	48.73	0.32
M4	poriferast-5-en-3beta-ol	414.79	1	1	36.91	0.75
M3	beta-sitosterol	414.79	1	1	36.91	0.75
M6	Stigmasterol	412.77	1	1	43.83	0.76
M12	Glypallichalcone	284.33	1	4	61.60	0.19

DL = drug-likeness, Hacc = hydrogen acceptors, Hdon = hydrogen donors, ID = identification, MW = molecule weight, OB = oral bioavailability, ZOR = *Zingiber officinale* Roscoe.

### 3.2. Screening of potential targets and acquisition of hub genes

**Figure 1.**
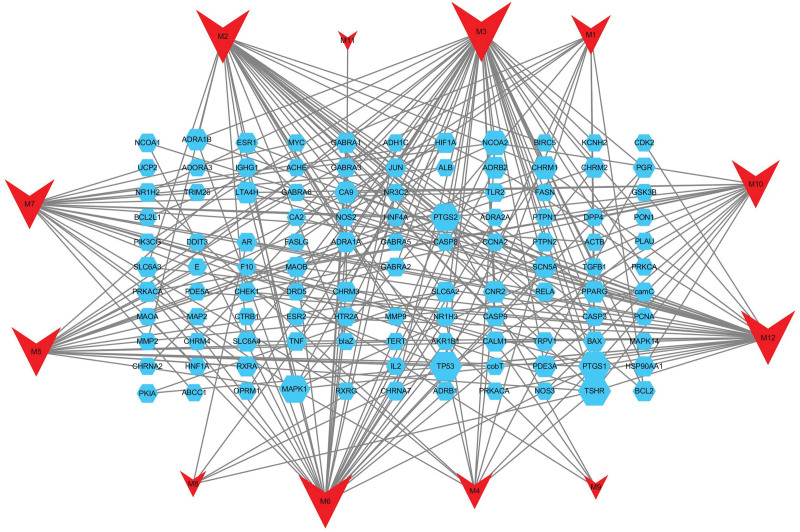
Active compounds and their corresponding target network of ZOR. Red indicates the compounds, blue indicates the target. Compound and protein nodes are linked if the corresponding compound targets the protein. The node size is proportional to its degree. ZOR = *Zingiber officinale* Roscoe.

**Figure 2.**
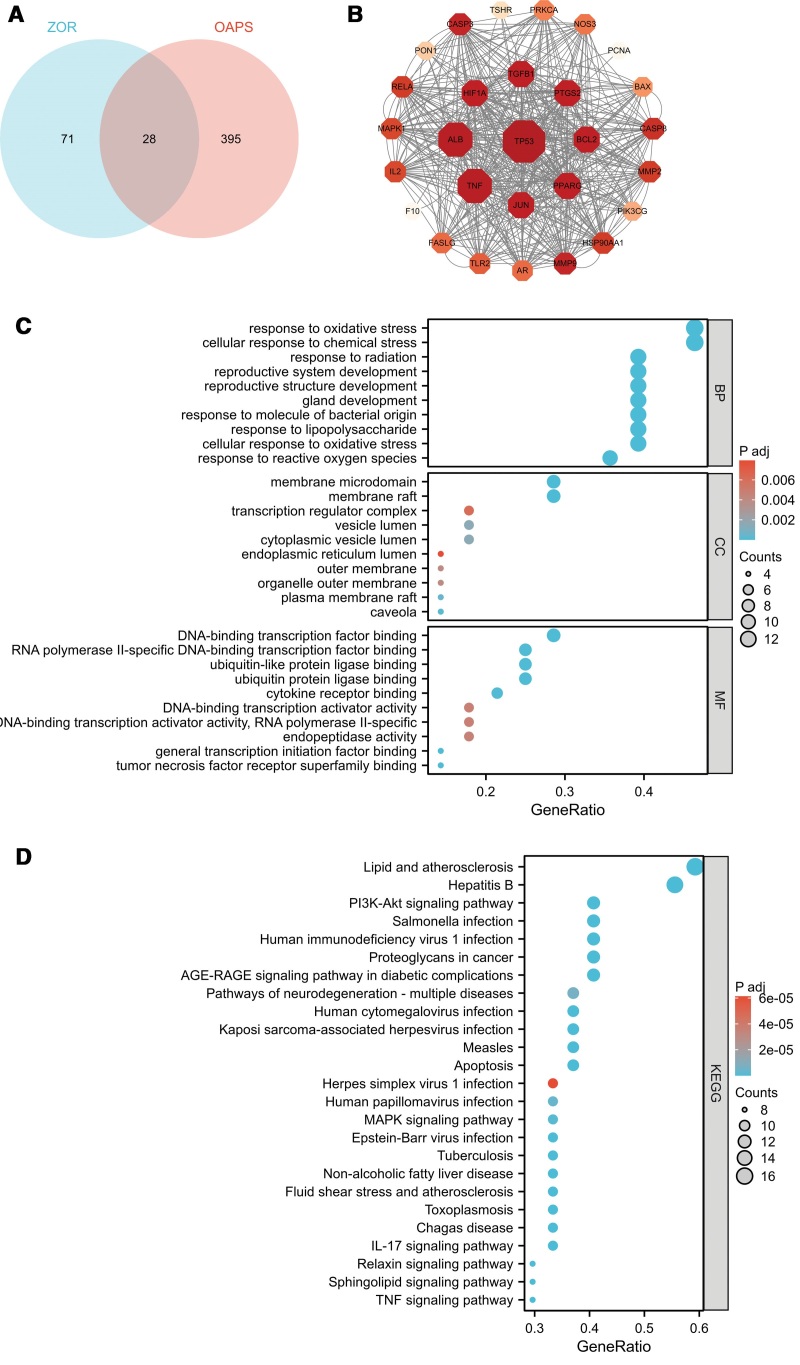
Interactions and functions of ZOR-associated targets in OAPS. (A) Intersecting targets of ZOR and OAPS. (B) The PPI network of the 28 hub genes. The larger the node and the redder its color, the higher the degree value of the corresponding target. Lines indicate that 2 targets are associated. (C) The Y-axis shows the top 10 for GO terms (BP, CC and MF), and the X-axis shows the enrichment scores of these terms. (D) The Y-axis shows the top 25 for KEGG enrichment terms, and the X-axis shows the enrichment scores of these terms. BP = biological process, CC = cell component, GO = Gene Ontology, KEGG = Kyoto Encyclopedia of Genes and Genomes, MF = molecular function, OAPS = obstetric antiphospholipid syndrome, PPI = protein–protein interaction, TNF = tumor necrosis factor, ZOR = *Zingiber officinale* Roscoe.

We identified 99 potential candidate targets (Appendix 1, Supplemental Digital Content), each of which corresponded to 12 active compounds. Based on these targets and compounds, a C-T network (Fig. 1) was established with Cytoscape 3.10.3. According to the C-T interactions, the average target number of each compound was 9.167, which could explain the synergistic or additive effects of ZOR in protecting OAPS. In total, 423 OAPS-related targets were obtained from GeneCards (Appendix 2, Supplemental Digital Content). A total of 28 common targets were obtained from 99 ZOR-related targets and 423 OAPS-related targets by Venn diagram analysis (Fig. 2A), which were selected as hub genes in subsequent analysis.

### 3.3. Construction of target protein interaction network and GO, KEGG enrichment

To clarify the functions of the 28 hub genes in OAPS, we conducted PPI, GO, and KEGG analyses. A PPI network (Fig. 2B) was constructed using Cytoscape software. In this network, nodes represent proteins, and edges represent interrelationships. The top 9 genes, serving as key nodes in the network, include tumor protein 53 (TP53), tumor necrosis factor (TNF), serum albumin, prostaglandin G/H synthase 2 (PTGS2), apoptosis regulator Bcl-2, transcription factor AP-1, hypoxia-inducible factor 1-alpha, peroxisome proliferator-activated receptor gamma, and transforming growth factor beta-1. These hub genes were mainly involved in the analysis of 1898 BPs, 45 CCs, and 146 MFs. The top 10 terms are displayed as bubble charts (Fig. 2C).

**Figure 3.**
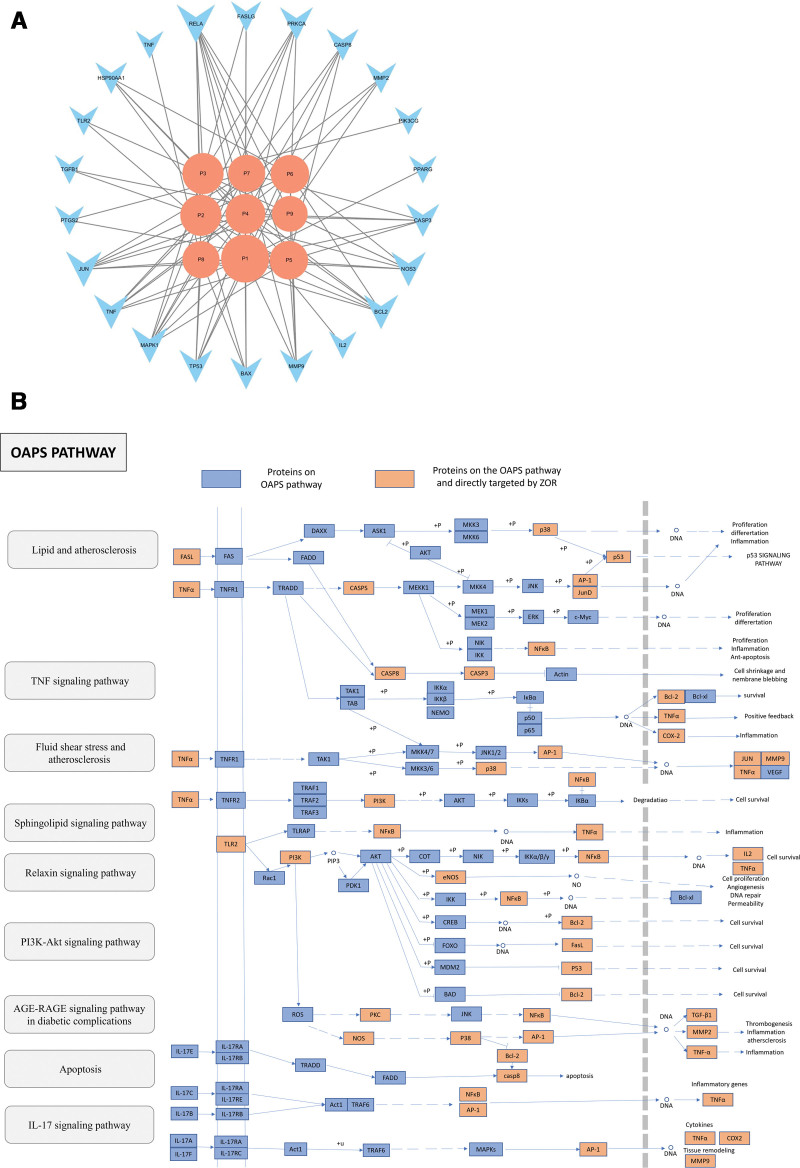
OAPS pathway and its key targets. (A) T-P network of hub genes: The node size is proportional to its degree. The pathway information was obtained by mapping the target proteins to the KEGG pathway database. (B) Distribution of the target proteins of ZOR on a compressed “OAPS pathway.” Nine pathways (gray) form the compressed OAPS pathway. Arrows represent activation effect, T-arrows represent inhibition effect, and segments show activation effect or inhibition effect. KEGG = Kyoto Encyclopedia of Genes and Genomes, OAPS = obstetric antiphospholipid syndrome, T-P = target-pathway, TNF = tumor necrosis factor, ZOR = *Zingiber officinale* Roscoe.

We obtained 131 pathways after repeated analysis and further screened 69 pathways with degrees of >5 and *P* values of <.01, which could be used in future studies investigating the use of ZOR in disease protection. According to the *P*-value, the top 25 are displayed as bubble plots (Fig. 2D). We screened 9 OAPS-related pathways (Table 2) based on the OAPS-related BP, MF, and CC. A T-P network (Fig. 3A) was established, and 21 targets were further mapped to 9 pathways, which might be important factors in the development of OAPS. Furthermore, 21 of the 28 target proteins were mapped to multiple pathways. The key pathways identified via KEGG and T-P network analyses were combined to form an integrated “OAPS pathway” (Fig. 3B). The “OAPS pathway” target proteins had an obvious functional relationship with OAPS-related proteins and had 3 representative therapeutic modules: anticoagulation, anti-inflammation, and immunoregulation.

**Table 2:** Nine pathways and their targets in ZOR and OAPS.

ID	Pathway	Targets	*P* values
P1	Lipid and atherosclerosis (hsa05417)	BAX, BCL2, CASP3, CASP8, FASLG, HSP90AA1, JUN, MAPK1, MMP9, NOS3, PPARG, PRKCA, RELA, TLR2, TNF, TP53	3.09463E−19
P2	AGE-RAGE signaling pathway in diabetic complications (hsa04933)	BAX, BCL2, CASP3, JUN, MAPK1, MMP2, NOS3, PRKCA, RELA, TGFB1, TNF	5.88311E−15
P3	PI3K-Akt signaling pathway (hsa04151)	BCL2, FASLG, HSP90AA1, IL2, MAPK1, NOS3, PIK3CG, PRKCA, RELA, TLR2, TP53	6.09781E−09
P4	Apoptosis (hsa04210)	BAX, BCL2, CASP3, CASP8, FASLG, JUN, MAPK1, RELA, TNF, TP53	7.82157E−12
P5	IL-17 signaling pathway (hsa04657)	CASP3, CASP8, HSP90AA1, JUN, MAPK1, MMP9, PTGS2, RELA, TNF	9.51816E−12
P6	Fluid shear stress and atherosclerosis (hsa05418)	BCL2, HSP90AA1, JUN, MMP2, MMP9, NOS3, RELA, TNF, TP53	3.34985E−10
P7	TNF signaling pathway (hsa04668)	CASP3, CASP8, JUN, MAPK1, MMP9, PTGS2, RELA, TNF	1.74287E−09
P8	Sphingolipid signaling pathway (hsa04071)	BAX, BCL2, MAPK1, NOS3, PRKCA, RELA, TNF, TP53	2.83279E−09
P9	Relaxin signaling pathway (hsa04926)	JUN, MAPK1, MMP2, MMP9, NOS3, PRKCA, RELA, TGFB1	5.39167E−09

ALB = serum albumin, AGE = advanced glycation end product, BAX = apoptosis regulator BAX, BCL2 = apoptosis regulator Bcl-2, CASP3 = caspase-3, CASP8 = caspase-8, FASLG = tumor necrosis factor ligand superfamily member 6, HSP90AA1 = heat shock protein HSP 90-alpha, JUN = transcription factor AP-1, IL2 = interleukin 2, MAPK1 = mitogen-activated protein kinase 1, MMP2 = 72 kDa type IV collagenase, MMP9 = matrix metalloproteinase-9, NOS3 = nitric oxide synthase endothelial 3, PIK3CG = phosphatidylinositol 4,5-bisphosphate 3-kinase catalytic subunit gamma isoform, PPARG = peroxisome proliferator-activated receptor gamma, PRKCA = protein kinase C alpha type, PTGS2 = prostaglandin G/H synthase 2, RAGE = receptor for advanced glycation end product, RELA = transcription factor p65, TGFB1 = transforming growth factor beta-1, TLR2 = toll-like receptor 2, TNF = tumor necrosis factor, TP53 = tumor protein 53, ZOR = *Zingiber officinale* Roscoe.

### 3.4. Molecular docking

**Figure 4.**
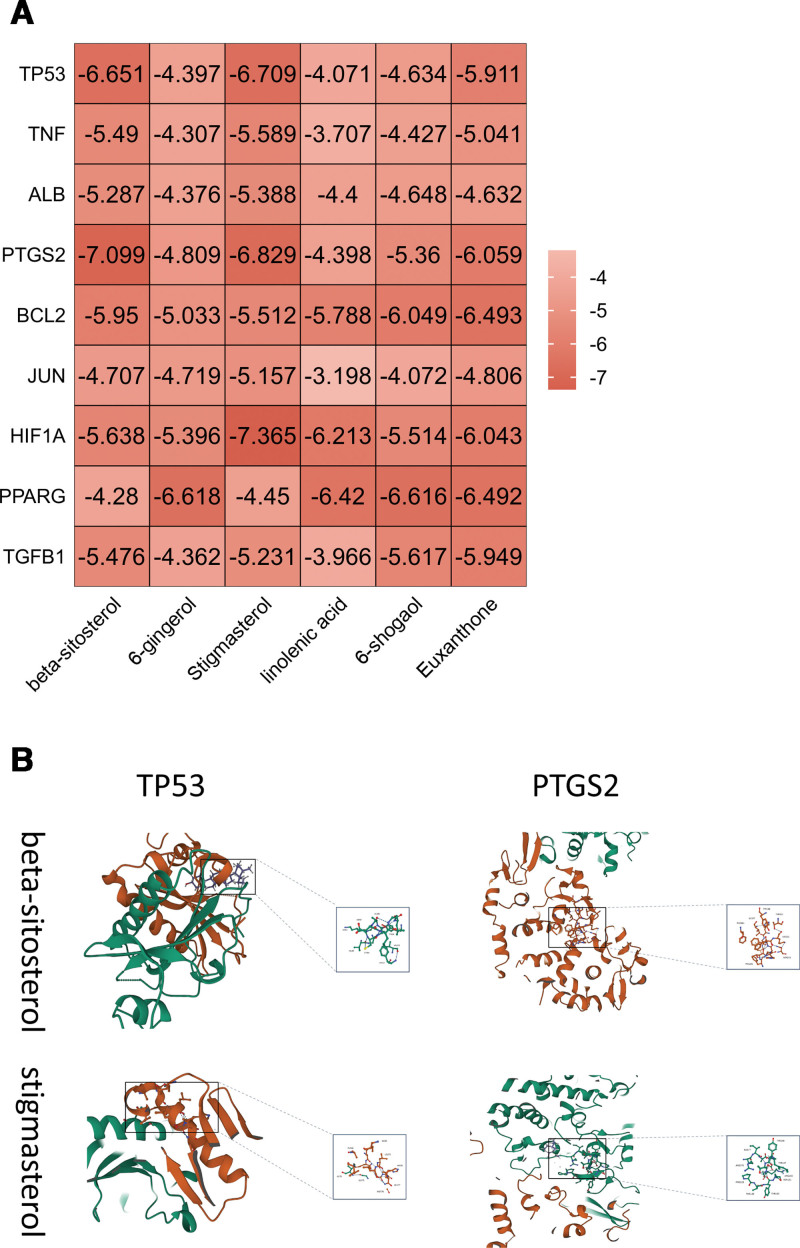
Molecular docking verification results. (A) Docking results of core active ingredients and key node proteins. (B) Molecular docking results of TP53 with beta-sitosterol, TP53 with stigmasterol, PTGS2 with beta-sitosterol, and PTGS2 with stigmasterol. AKT = protein kinase B, ALB = serum albumin, BCL2 = apoptosis regulator Bcl-2, JUN = transcription factor AP-1, OAPS = obstetric antiphospholipid syndrome, PI3K = phosphatidylinositol 3-kinase, PPARG = peroxisome proliferator-activated receptor gamma, PTGS2 = prostaglandin G/H synthase 2, TGFB1 = transforming growth factor beta-1, TNF = tumor necrosis factor, TP53 = tumor protein 53, ZOR = *Zingiber officinale* Roscoe.

Molecular docking simulates the recognition of drug molecules and protein targets at the atomic level. Among the active ingredients of ZOR, we selected beta-sitosterol, 6-gingerol, stigmasterol, linolenic acid, 6-shogaol, and euxanthone as ligands for molecular docking with TP53, TNF, serum albumin, PTGS2, apoptosis regulator Bcl-2, transcription factor AP-1, hypoxia-inducible factor 1-alpha, peroxisome proliferator-activated receptor gamma, and transforming growth factor beta-1 (Fig. 4A). The smaller the binding energy required when the ligand is docked with the receptor molecule, the easier it is to bind, and the more stable is the structure formed. Our results showed that the binding affinities of TP53 with beta-sitosterol, TP53 with stigmasterol, PTGS2 with beta-sitosterol, and PTGS2 with stigmasterol were −6.651, −6.709, −7.099, and −6.829, respectively, all of which had high binding affinities (Fig. 4B).

## 4. Discussion

APS is prone to complications such as fetal loss and recurrent thrombosis during pregnancy. Anticoagulation is currently the main treatment measure, which can improve the probability of maintaining pregnancy. In recent years, immunotherapy and anti-inflammatory therapy have become hotspots in research. As a medicinal herb with both food and medicinal properties, ZOR has anti-inflammatory and immunomodulatory effects, but its application in obstetrics remains unexplored. Based on systems pharmacology and molecular docking technology, this study explores the effective compounds and their targets of ZOR, and discusses the anticoagulant, anti-inflammatory, and immunomodulatory effects of ZOR in the protection against OAPS.

In our study, 12 effective compounds were identified in ZOR. 6-gingerol (M2) is the major component and has been confirmed to possess anti-inflammatory,^[10,11]^ antioxidant,^[3,12]^ antiangiogenic,^[5]^ antihyperglycemic,^[13]^ antiemetic,^[14]^ and antitumor activities.^[12]^ Stigmasterol (M6) has numerous pharmacological effects, including anti-inflammatory,^[15]^ antioxidation,^[16]^ and antitumor properties. Beta-sitosterol is an important constituent of ZOR, and experimental studies have reported that it can be used to improve the prognosis of osteoporosis and its antioxidative properties.^[17]^ Beta-sitosterol demonstrated its anti-inflammatory prowess by effectively curbing the expression of key inflammatory cytokines in lipopolysaccharide-treated human umbilical vein endothelial cells. Specifically, it managed to reduce the levels of TNF-α, cyclooxygenase-2 (COX-2), and interleukin (IL)-6.^[18]^ Dihydrocapsaicin, the main component of capsicum, is an active ingredient in ZOR and has antioxidant properties.^[19]^ Recent findings have shown that the combination of dihydrocapsaicin and capsaicin inhibited platelet aggregation and thromboxane formation in vitro.^[20]^

Our findings show that 1 target can be simultaneously targeted by multiple compounds. PTGS1 (degree = 9) showed the highest degree. When PTGS1 is activated, it produces many prostaglandins that are involved in anticoagulation.^[21]^ PTGS2 (COX-2; degree = 8) is associated with inflammation,^[22]^ and COX-2 expression in platelets plays an important role in tumorigenesis and thrombosis.^[23]^ TNF (degree = 2) is a multifunctional cytokine that is involved in various important physiological processes, such as apoptosis and survival, inflammatory reaction, and autoimmunity. These results suggest that the various drug targets may be associated with OAPS protection.

It has been reported that APL can cause thrombosis by endothelial activation, affecting the coagulation system, complement activation, and secretion of cytokines and other inflammatory mediators.^[24]^ OAPS is believed to be caused by placental vascular involvement. Moreover, although noninflammatory vascular thrombosis is an important feature of APS, reliable evidence of inflammation and complement activation has been obtained in the placental tissue of an APL-induced APS mouse model.^[25]^ Therefore, our findings suggest that the nonexclusive association of anticoagulation, anti-inflammation, and autoimmune regulation is the main strategy for protection against OAPS.

Placental blood vessels are systemic blood vessels, and placental blood clots are found in approximately one-third of women with OAPS. Furthermore, nitric oxide is a gas that dilates blood vessels and inhibits platelet aggregation. Through phosphatidylinositol 3-kinase/protein kinase B (AKT)/endothelial nitric oxide synthasesignaling pathway activation, 6-gingerol was shown to substantially prevent the decrease in nitric oxide production and attenuate the injury of human umbilical vein endothelial cells.^[26]^ The potential use of 6-gingerol and 6-shogaol as COX-2 inhibitors has been reported,^[27]^ which can, in turn, inhibit platelet aggregation.

In patients with OAPS, the levels of neutrophil extracellular traps (NETs) increase during the early stages of pregnancy. APLAs inhibit trophoblast migration and invasion by increasing reactive oxygen species production and phosphorylation of AKT and extracellular regulated protein kinases, which can also impair endothelial migration and angiogenesis. These factors affect the placental formation process and cause pregnancy-related complications. Several studies have shown that 6-gingerol can suppress NETs formation in response to various stimuli, including OAPS-related stimuli.^[11]^ Other studies have shown that the activation of AKT, ERK1/2, and p38 mitogen-activated protein kinase pathways promotes the release of NETs, but they are not the only pathway.^[28]^ Thus, in protecting OAPS, 6-gingerol may inhibit NET formation via the phosphatidylinositol 3-kinase/AKT signaling pathway and act as an anti-inflammatory agent. In addition, 6-gingerol derivatives play an antioxidant role by acting as free radical scavengers or xanthine oxidase inhibitors.^[29]^

IL-17 is a pro-inflammatory cytokine that is mainly secreted by T-helper (Th)17 cells. Studies have shown that stimulation of the IL-23/IL-17 axis by TGFβ increases IL-17A gene polymorphism and APL production in primary APS.^[30]^ In the current study, 6-gingerol significantly alleviated the inflammatory injury of the intestinal tract in a mouse model by reducing the level of IL-17 in serum and intestinal tissue and inhibiting the phosphorylation levels of IκBα and p65.^[31]^ Studies have shown that elevated concentrations of APLAs can result in an increased frequency of circulating Th2 and Th17 cells in OAPS.^[32]^ Furthermore, it has been shown that in patients with typical and non-criteria OAPS, differentially expressed genes are upregulated in pathways related to Th1, Th2, and Th17 cell differentiation, which are prominently manifested in OAPS patients.^[33]^ IL-1 and TNF-α enhance the polarization and activation of Th17 cells,^[34]^ whereas 6-gingerol inhibits the lipopolysaccharide-mediated expression of COX-2, IL-1β, and TNF-α by downregulating MAPK signal transduction and transcription factor p65 activation in microglial cells,^[35]^ indicating that Th17 cells are related to the 6-gingerol inhibition response. ZOR also inhibits the production of IL-6, which reduces Th17 cell-related responses.^[36]^ Furthermore, 6-gingerol was shown to inhibit the production of Th1- and Th2-type cytokines in ovalbumin-stimulated splenocytes as well as the expansion and differentiation of Th1 and Th2 cells from lymphocytes.^[37]^ The study demonstrated the anti-asthmatic effects of 6-shogaol and 6-gingerol on the Th2 cell-mediated allergic response pathway in an ovalbumin-induced asthma mouse model.^[38]^ Therefore, the immunomodulatory effects of 6-gingerol are involved in Th2 cells in various diseases. Overall, the findings of this study, along with corroborating evidence from literature reports, may provide a foundation for selecting potential molecules that can be used for the protection of OAPS. Our research is based on computer data analysis. However, while computer data analysis provides valuable preliminary findings and hypotheses, these results require further experimental verification to confirm their validity and generalizability.

## 5. Conclusion

Our results provide a novel approach, and at the same time identify the potential molecular targets and signaling pathways of ZOR relevant to OAPS. These findings contribute valuable insights into the mechanistic basis of ZOR’s effects; however, further in vitro experiments and animal model studies are necessary to validate and confirm these preliminary observations.

## Acknowledgments

We also thank Editage (www.editage.cn) for English language editing.

## Author contributions

**Funding acquisition:** Hui Li.

**Data curation:** Bo Du, Qun Wang, Ning He, Hui Li.

**Formal analysis:** Bo Du, Hongmei Wang.

**Project administration:** Bo Du, Qun Wang.

**Software:** Bo Du, Ning He.

**Validation:** Hongmei Wang, Kai Wang.

**Writing – original draft:** Bo Du, Hongmei Wang, Hui Li.

**Writing – review & editing:** Bo Du, Hui Li.





## References

[1] Knight JS Branch DW Ortel TL. Antiphospholipid syndrome: advances in diagnosis, pathogenesis, and management. BMJ. 2023;380:e069717. 36849186 10.1136/bmj-2021-069717

[2] Murvai VR Galiș R Panaitescu A. Antiphospholipid syndrome in pregnancy: a comprehensive literature review. BMC Pregnancy Childbirth. 2025;25:337–54. 40128683 10.1186/s12884-025-07471-w PMC11934569

[3] Mustafa I Chin NL. Antioxidant properties of dried ginger (Zingiber officinale Roscoe) var. Bentong. Foods. 2023;12:178–95. 36613394 10.3390/foods12010178 PMC9818862

[4] Ayustaningwarno F Anjani G Ayu AM Fogliano V. A critical review of ginger’s (Zingiber officinale) antioxidant, anti-inflammatory, and immunomodulatory activities. Front Nutr. 2024;11:1364836–51. 38903613 10.3389/fnut.2024.1364836 PMC11187345

[5] Ma H Li J. The ginger extract could improve diabetic retinopathy by inhibiting the expression of e/iNOS and G6PDH, apoptosis, inflammation, and angiogenesis. J Food Biochem. 2022;46:e14084. 35060143 10.1111/jfbc.14084

[6] Pázmándi K Szöllősi AG Fekete T. The “root” causes behind the anti-inflammatory actions of ginger compounds in immune cells. Front Immunol. 2024;15:1400956. 39007134 10.3389/fimmu.2024.1400956 PMC11239339

[7] Ru J Li P Wang J. TCMSP: a database of systems pharmacology for drug discovery from herbal medicines. J Cheminform. 2014;6:13. 24735618 10.1186/1758-2946-6-13 PMC4001360

[8] Zheng C Guo Z Huang C. Large-scale direct targeting for drug repositioning and discovery. Sci Rep. 2015;5:11970. 26155766 10.1038/srep11970 PMC4496667

[9] Bugnon M Röhrig UF Goullieux M. SwissDock 2024: major enhancements for small-molecule docking with Attracting Cavities and AutoDock Vina. Nucleic Acids Res. 2024;52:W324–32. 38686803 10.1093/nar/gkae300 PMC11223881

[10] Mchugh J. Getting to the root of the anti-inflammatory effects of ginger. Nat Rev Rheumatol. 2021;17:130. 10.1038/s41584-021-00577-3 33504929

[11] Ali RA Gandhi AA Dai L Weiner JK Knight JS. Anti-neutrophil properties of natural gingerols in models of lupus. JCI Insight. 2020;6:e138385. 10.1172/jci.insight.138385 PMC7934838 33373329

[12] Wu Z Gao R Li H. New insight into the joint significance of dietary jujube polysaccharides and 6-gingerol in antioxidant and antitumor activities. RSC Adv. 2021;11:33219–34. 35497558 10.1039/d1ra03640h PMC9042247

[13] Singh KK Mangang HA Singh OK. Comprehensive metabolomic and bioactivity profiling of Zingiberaceae species from Manipur: elucidating antidiabetic and antioxidant mechanisms through in vitro and in silico approaches. Phytochem Anal. 2025;36:1396–415. 39993938 10.1002/pca.3517

[14] Cheng Q Feng X Meng Q. [6]-Gingerol ameliorates cisplatin-induced pica by regulating the TPH/MAO-A/SERT/5-HT/5-HT3 receptor system in rats. Drug Des Devel Ther. 2020;14:4085–99. 10.2147/DDDT.S270185 PMC7538004 33061309

[15] Morgan LV Petry F Scatolin M. Investigation of the anti-inflammatory effects of stigmasterol in mice: insight into its mechanism of action. Behav Pharmacol. 2021;32:640–51. 34657071 10.1097/FBP.0000000000000658

[16] Ohara Y Osada K. Effect of dietary oxidized stigmasterol on the antioxidant system in mice. J Oleo Sci. 2024;73:1493–503. 39617431 10.5650/jos.ess24167

[17] Parvez MK Alam P Arbab AH Al-Dosari MS Alhowiriny TA Alqasoumi SI. Analysis of antioxidative and antiviral biomarkers β-amyrin, β-sitosterol, lupeol, ursolic acid in Guiera senegalensis leaves extract by validated HPTLC methods. Saudi Pharm J. 2018;26:685–93. 29991912 10.1016/j.jsps.2018.02.022 PMC6035322

[18] Bi Y Liang H Han X. β-Sitosterol suppresses LPS-induced cytokine production in human umbilical vein endothelial cells via MAPKs and NF-κB signaling pathway. Evid Based Complement Alternat Med. 2023;2023:9241090. 36636603 10.1155/2023/9241090 PMC9831711

[19] Janyou A Wicha P Seechamnanturakit V. Dihydrocapsaicin-induced angiogenesis and improved functional recovery after cerebral ischemia and reperfusion in a rat model. J Pharmacol Sci. 2020;143:9–16. 32107104 10.1016/j.jphs.2020.02.001

[20] Almaghrabi S Adams M Geraghty D Ahuja K. Synergistic inhibitory effect of capsaicin and dihydrocapsaicin on in-vitro platelet aggregation and thromboxane formation. Blood Coagul Fibrinolysis. 2018;29:351–5. 29634579 10.1097/MBC.0000000000000698

[21] Xiang B Zhang G Guo L. Platelets protect from septic shock by inhibiting macrophage-dependent inflammation via the cyclooxygenase 1 signalling pathway. Nat Commun. 2013;4:2657–84. 24150174 10.1038/ncomms3657 PMC4217311

[22] Cui J Jia J. Natural COX-2 inhibitors as promising anti-inflammatory agents: an update. Curr Med Chem. 2021;28:3622–46. 32942970 10.2174/0929867327999200917150939

[23] Hu Q Cho M Thiagarajan P Aung FM Sood AK Afshar-Kharghan V. A small amount of cyclooxygenase 2 (COX2) is constitutively expressed in platelets. Platelets. 2016;28:99–102. 27534811 10.1080/09537104.2016.1203406 PMC5310196

[24] Raschi E Borghi MO Tedesco F Meroni PL. Antiphospholipid syndrome pathogenesis in 2023: an update of new mechanisms or just a reconsideration of the old ones? Rheumatology (Oxford). 2024;63:SI4–SI13. 38320591 10.1093/rheumatology/kead603

[25] Wang C Li A Zhang C. Neutrophil extracellular traps aggravate placental injury in OAPS by facilitating activation of BNIP3 mediated mitophagy. Free Radic Biol Med. 2025;235:109–23. 40286883 10.1016/j.freeradbiomed.2025.04.038

[26] Liu D Wu M Lu Y Tao X Huang Q. Protective effects of 6-gingerol on vascular endothelial cell injury induced by high glucose via activation of PI3K-AKT-eNOS pathway in human umbilical vein endothelial cells. Biomed Pharmacother. 2017;93:788–95. 28709132 10.1016/j.biopha.2017.07.037

[27] Saptarini NM Itorus EY Levita J. Structure-based in silico study of 6-gingerol, 6-ghogaol, and 6-paradol, active compounds of ginger (zingiber officinale) as cox-2 inhibitors. Int J Chem. 2013;5:12–8.

[28] Lu Y Dong Y Zhang Y. Antiphospholipid antibody-activated NETs exacerbate trophoblast and endothelial cell injury in obstetric antiphospholipid syndrome. J Cell Mol Med. 2020;12:6690–703. 10.1111/jcmm.15321 PMC7299718 32369873

[29] Ahmed SHH Gonda T Agbadua OG. Preparation and evaluation of 6-gingerol derivatives as novel antioxidants and antiplatelet agents. Antioxidants (Basel). 2023;12:744. 36978992 10.3390/antiox12030744 PMC10045534

[30] Popovic-Kuzmanovic D Novakovic I Stojanovich L. Increased activity of interleukin-23/interleukin-17 cytokine axis in primary antiphospholipid syndrome. Immunobiology. 2013;218:186–91. 22559912 10.1016/j.imbio.2012.03.002

[31] Sheng Y Wu T Dai Y. 6-gingerol alleviates inflammatory injury in DSS-induced ulcerative colitis mice by regulating NF-κB signaling. Ann Palliat Med. 2020;9:1944–52. 32692218 10.21037/apm-20-903

[32] Jing X Zhu F Liu X Xiong J. Th1/Th2/Th17/Treg expression in cultured PBMCs with antiphospholipid antibodies. Mol Med Rep. 2012;6:1035–9. 22941119 10.3892/mmr.2012.1055

[33] Jadidi-Niaragh F Mirshafiey A. Th17 cell, the new player of neuroinflammatory process in multiple sclerosis. Scand J Immunol. 2011;74:1–13. 21338381 10.1111/j.1365-3083.2011.02536.x

[34] Qi X Liu P Zhou Y. Transcriptomics analysis of differential gene expression and immune and inflammatory response mechanisms in patients with typical and non-criteria obstetric antiphospholipid syndrome (OAPS and NC-OAPS). J Reprod Immunol. 2024;166:104389. 39522423 10.1016/j.jri.2024.104389

[35] Ha SK Moon E Ju MS. 6-shogaol, a ginger product, modulates neuroinflammation: a new approach to neuroprotection. Neuropharmacology. 2012;63:211–23. 22465818 10.1016/j.neuropharm.2012.03.016

[36] Ho SC Chang KS Lin CC. Anti-neuroinflammatory capacity of fresh ginger is attributed mainly to 10-gingerol. Food Chem. 2013;141:3183–91. 23871076 10.1016/j.foodchem.2013.06.010

[37] Kawamoto Y Ueno Y Nakahashi E. Prevention of allergic rhinitis by ginger and the molecular basis of immunosuppression by 6-gingerol through T cell inactivation. J Nutr Biochem. 2016;27:112–22. 26403321 10.1016/j.jnutbio.2015.08.025

[38] Kim E Jang S Yi JK. Ginger-derived compounds exert in vivo and in vitro anti-asthmatic effects by inhibiting the T-helper 2 cell-mediated allergic response. Exp Ther Med. 2022;23:49. 34934427 10.3892/etm.2021.10971 PMC8652391

