# Supplementary material for: Protective effects of Zingiber officinale Roscoe in obstetric antiphospholipid syndrome based on systems pharmacology and molecular docking
Source: Medicine (Baltimore). 2026 May 8;105(19):e48706. doi: 10.1097/MD.0000000000048706 (PMC13166589; doi:10.1097/MD.0000000000048706)
Supplement: Supplementary file 1 [file medi-105-e48706-s001.doc]

Appendix 1 99 potential targets and their parameters

| **UniProt ID** | | **Description** | **Gene Symbol** | **Species** |
| --- | --- | --- | --- | --- |
| P06401 | Progesterone receptor | | PGR | Homo sapiens (Human) |
| Q15596 | Nuclear receptor coactivator 2 | | NCOA2 | Homo sapiens (Human) |
| P23219 | Prostaglandin G/H synthase 1 | | PTGS1 | Homo sapiens (Human) |
| P35354 | Prostaglandin G/H synthase 2 | | PTGS2(COX2) | Homo sapiens (Human) |
| P07900 | Heat shock protein HSP 90 | | HSP90AA1 | Homo sapiens (Human) |
| P48736 | Phosphatidylinositol-4,5-bisphosphate3-kinase catalytic subunit, gamma isoform | | PIK3CG | Homo sapiens (Human) |
| Q12809 | Potassium voltage-gated channel subfamily H member 2 | | KCNH2 | Homo sapiens (Human) |
| [P17612](http://www.uniprot.org/uniprot/P17612) | mRNA of PKA Catalytic Subunit C-alpha | | PRKACA | Homo sapiens (Human) |
| [P21918](https://www.uniprot.org/uniprot/P21918) | Dopamine D1 receptor | | DRD5 | Homo sapiens (Human) |
| P20309 | Muscarinic acetylcholine receptor M3 | | CHRM3 | Homo sapiens (Human) |
| P11229 | Muscarinic acetylcholine receptor M1 | | CHRM1 | Homo sapiens (Human) |
| Q14524 | Sodium channel protein type 5 subunit alpha | | SCN5A | Homo sapiens (Human) |
| [P47869](http://www.uniprot.org/uniprot/P47869) | Gamma-aminobutyric-acid receptor alpha-2 subunit | | GABRA2 | Homo sapiens (Human) |
| P08173 | Muscarinic acetylcholine receptor M4 | | CHRM4 | Homo sapiens (Human) |
| Q14432 | CGMP-inhibited 3',5'-cyclic phosphodiesterase A | | PDE3A | Homo sapiens (Human) |
| P28223 | 5-hydroxytryptamine 2A receptor | | HTR2A | Homo sapiens (Human) |
| [P31644](https://www.uniprot.org/uniprot/P31644) | Gamma-aminobutyric-acid receptor alpha-5 subunit | | GABRA5 | Homo sapiens (Human) |
| P35348 | Alpha-1A adrenergic receptor | | ADRA1A | Homo sapiens (Human) |
| [P34903](https://www.uniprot.org/uniprot/P34903) | Gamma-aminobutyric-acid receptor alpha-3 subunit | | GABRA3 | Homo sapiens (Human) |
| P08172 | Muscarinic acetylcholine receptor M2 | | CHRM2 | Homo sapiens (Human) |
| P35368 | Alpha-1B adrenergic receptor | | ADRA1B | Homo sapiens (Human) |
| P07550 | Beta-2 adrenergic receptor | | ADRB2 | Homo sapiens (Human) |
| Q15822 | Neuronal acetylcholine receptor subunit alpha-2 | | CHRNA2 | Homo sapiens (Human) |
| P31645 | Sodium-dependent serotonin transporter | | SLC6A4 | Homo sapiens (Human) |
| P35372 | Mu-type opioid receptor | | OPRM1 | Homo sapiens (Human) |
| [P14867](http://www.uniprot.org/uniprot/P14867) | Gamma-aminobutyric acid receptor subunit alpha-1 | | GABRA1 | Homo sapiens (Human) |
| [P36544](https://www.uniprot.org/uniprot/P36544) | Neuronal acetylcholine receptor protein, alpha-7 chain | | CHRNA7 | Homo sapiens (Human) |
| P00183 | Cytochrome P450-cam | | CAMC | Homo sapiens (Human) |
| P10415 | Apoptosis regulator Bcl-2 | | BCL2 | Homo sapiens (Human) |
| [Q07812](http://www.uniprot.org/uniprot/Q07812) | Apoptosis regulator BAX | | BAX | Homo sapiens (Human) |
| [P55211](http://www.uniprot.org/uniprot/P55211) | Caspase-9 | | CASP9 | Homo sapiens (Human) |
| P05412 | Transcription factor AP-1 | | JUN | Homo sapiens (Human) |
| P42574 | Caspase-3 | | CASP3 | Homo sapiens (Human) |
| [Q14790](http://www.uniprot.org/uniprot/Q14790) | Caspase-8 | | CASP8 | Homo sapiens (Human) |
| P17252 | Protein kinase C alpha type | | PRKCA | Homo sapiens (Human) |
| P01137 | Transforming growth factor beta-1 | | TGFB1 | Homo sapiens (Human) |
| P27169 | Serum paraoxonase/arylesterase 1 | | PON1 | Homo sapiens (Human) |
| P11137 | Microtubule-associated protein 2 | | MAP2 | Homo sapiens (Human) |
| [P34972](https://www.uniprot.org/uniprot/P34972) | cannabinoid receptor 2 | | CNR2 | Homo sapiens (Human) |
| P16473 | Thyrotropin receptor | | TSHR | Homo sapiens (Human) |
| [Q13133](https://www.uniprot.org/uniprot/Q13133) | Oxysterols receptor LXR-alpha | | NR1H3 | Homo sapiens (Human) |
| [P55055](https://www.uniprot.org/uniprot/P55055) | Oxysterols receptor LXR-beta | | NR1H2 | Homo sapiens (Human) |
| [P04637](https://www.uniprot.org/uniprot/P04637) | P53 family | | TP53 | Homo sapiens (Human) |
| P28482 | Mitogen-activated protein kinase 1 | | MAPK1 | Homo sapiens (Human) |
| P17706 | Tyrosine-protein phosphatase non-receptor type 2 | | PTPN2 | Homo sapiens (Human) |
| P01375 | Tumor necrosis factor | | TNF | Homo sapiens (Human) |
| P08235 | Mineralocorticoid receptor | | NR3C2 | Homo sapiens (Human) |
| P00326 | Alcohol dehydrogenase 1C | | ADH1C | Homo sapiens (Human) |
| P01857 | Ig gamma-1 chain C region | | IGHG1 | Homo sapiens (Human) |
| P19793 | Retinoic acid receptor RXR-alpha | | RXRA | Homo sapiens (Human) |
| Q15788 | Nuclear receptor coactivator 1 | | NCOA1 | Homo sapiens (Human) |
| P08913 | Alpha-2A adrenergic receptor | | ADRA2A | Homo sapiens (Human) |
| P23975 | Sodium-dependent noradrenaline transporter | | SLC6A2 | Homo sapiens (Human) |
| Q01959 | Sodium-dependent dopamine transporter | | SLC6A3 | Homo sapiens (Human) |
| P15121 | Aldose reductase | | AKR1B1 | Homo sapiens (Human) |
| P00749 | Urokinase-type plasminogen activator | | PLAU | Homo sapiens (Human) |
| P09960 | Leukotriene A-4 hydrolase | | LTA4H | Homo sapiens (Human) |
| P27338 | Amine oxidase [flavin-containing] B | | MAOB | Homo sapiens (Human) |
| P21397 | Amine oxidase [flavin-containing] A | | MAOA | Homo sapiens (Human) |
| P17538 | Chymotrypsinogen B | | CTRB1 | Homo sapiens (Human) |
| P08588 | Beta-1 adrenergic receptor | | ADRB1 | Homo sapiens (Human) |
| P00720 | Lysozyme | | E | Homo sapiens (Human) |
| Q05603 | Nicotinate-nucleotide--dimethylbenzimidazole phosphoribosyltransferase | | cobT | Homo sapiens (Human) |
| Q8NER1 | Transient receptor potential cation channel subfamily V member 1 | | TRPV1 | Homo sapiens (Human) |
| Q16445 | Gamma-aminobutyric-acid receptor subunit alpha-6 | | GABRA6 | Homo sapiens (Human) |
| O14746 | Telomerase reverse transcriptase | | TERT | Homo sapiens (Human) |
| O60603 | Toll-like receptor 2 | | TLR2 | Homo sapiens (Human) |
| Q16665 | Hypoxia-inducible factor 1-alpha | | HIF1A | Homo sapiens (Human) |
| P48443 | Retinoic acid receptor RXR-gamma | | RXRG | Homo sapiens (Human) |
| [Q04206](http://www.uniprot.org/uniprot/Q04206) | Transcription factor p65 | | RELA | Homo sapiens (Human) |
| [P12004](http://www.uniprot.org/uniprot/P12004) | Proliferating cell nuclear antigen | | PCNA | Homo sapiens (Human) |
| P02768 | Serum albumin | | ALB | Homo sapiens (Human) |
| P01106 | Myc proto-oncogene protein | | MYC | Homo sapiens (Human) |
| P62158 | Calmodulin | | CALM1 | Homo sapiens (Human) |
| P41235 | Hepatocyte nuclear factor 4-alpha | | HNF4A | Homo sapiens (Human) |
| [P55851](https://www.uniprot.org/uniprot/P55851) | Mitochondrial uncoupling protein 2 | | UCP2 | Homo sapiens (Human) |
| P20823 | Hepatocyte nuclear factor 1-alpha | | HNF1A | Homo sapiens (Human) |
| [Q12899](https://www.uniprot.org/uniprot/Q12899) | Tripartite motif-containing protein 26 | | TRIM26 | Homo sapiens (Human) |
| P33527 | Multidrug resistance-associated protein 1 | | ABCC1 | Homo sapiens (Human) |
| [Q07817](http://www.uniprot.org/uniprot/Q07817) | Bcl-2-like protein 1 | | BCL2L1 | Homo sapiens (Human) |
| P49327 | Fatty acid synthase | | FASN | Homo sapiens (Human) |
| P37231 | Peroxisome proliferator-activated receptor gamma | | PPARG | Homo sapiens (Human) |
| [P48023](https://www.uniprot.org/uniprot/P48023) | Tumor necrosis factor ligand superfamily member 6 | | FASLG | Homo sapiens (Human) |
| [P35638](https://www.uniprot.org/uniprot/P35638) | DNA damage-inducible transcript 3 protein | | DDIT3 | Homo sapiens (Human) |
| Q16790 | Carbonic anhydrase 9 | | CA9 | Homo sapiens (Human) |
| P03372 | Estrogen receptor | | ESR1 | Homo sapiens (Human) |
| P22303 | Acetylcholinesterase | | ACHE | Homo sapiens (Human) |
| P00807 | Beta-lactamase | | blaZ | Homo sapiens (Human) |
| P08253 | 72 kDa type IV collagenase | | MMP2 | Homo sapiens (Human) |
| P28161 | Matrix metalloproteinase-9 | | MMP9 | Homo sapiens (Human) |
| [O15392](http://www.uniprot.org/uniprot/O15392) | Baculoviral IAP repeat-containing protein 5 | | BIRC5 | Homo sapiens (Human) |
| [P37231](http://www.uniprot.org/uniprot/P37231) | Peroxisome proliferator activated receptor gamma | | PPARG | Homo sapiens (Human) |
| P29474 | Nitric-oxide synthase, endothelial | | NOS3 | Homo sapiens (Human) |
| [P18031](https://www.uniprot.org/uniprot/P18031) | mRNA of Protein-tyrosine phosphatase, non-receptor type 1 | | PTPN1 | Homo sapiens (Human) |
| [P27487](https://www.uniprot.org/uniprot/P27487) | Dipeptidyl peptidase IV | | DPP4 | Homo sapiens (Human) |
| O14757 | Serine/threonine-protein kinase Chk1 | | CHEK1 | Homo sapiens (Human) |
| P61925 | cAMP-dependent protein kinase inhibitor alpha | | PKIA | Homo sapiens (Human) |
| P60568 | Interleukin-2 | | IL2 | Homo sapiens (Human) |
| P10275 | Androgen receptor | | AR | Homo sapiens (Human) |
| [P00742](http://www.uniprot.org/uniprot/P00742) | Coagulation factor Xa | | F10 | Homo sapiens (Human) |
| [P17612](https://www.uniprot.org/uniprot/P17612) | cAMP-dependent protein kinase catalytic subunit alpha | | PRKACA | Homo sapiens (Human) |
