# Supplementary material for: Protective effects of Zingiber officinale Roscoe in obstetric antiphospholipid syndrome based on systems pharmacology and molecular docking
Source: Medicine (Baltimore). 2026 May 8;105(19):e48706. doi: 10.1097/MD.0000000000048706 (PMC13166589; doi:10.1097/MD.0000000000048706)
Supplement: Supplementary file 2 [file medi-105-e48706-s002.docx]

Appendix 2 423 targets of OAPS from GeneCards

| Gene Symbol | Description | Category | Uniprot ID |
| --- | --- | --- | --- |
| TP53 | Tumor Protein P53 | Protein Coding | P04637 |
| PTEN | Phosphatase And Tensin Homolog | Protein Coding | P60484 |
| MECP2 | Methyl-CpG Binding Protein 2 | Protein Coding | P51608 |
| KMT2D | Lysine Methyltransferase 2D | Protein Coding | O14686 |
| CDH1 | Cadherin 1 | Protein Coding | P12830 |
| CREBBP | CREB Binding Protein | Protein Coding | Q92793 |
| PTPN11 | Protein Tyrosine Phosphatase Non-Receptor Type 11 | Protein Coding | Q06124 |
| NLRP3 | NLR Family Pyrin Domain Containing 3 | Protein Coding | Q96P20 |
| PIK3CA | Phosphatidylinositol-4,5-Bisphosphate 3-Kinase Catalytic Subunit Alpha | Protein Coding | P42336 |
| COL5A1 | Collagen Type V Alpha 1 Chain | Protein Coding | P20908 |
| APOH | Apolipoprotein H | Protein Coding | P02749 |
| F5 | Coagulation Factor V | Protein Coding | P12259 |
| CD40LG | CD40 Ligand | Protein Coding | P29965 |
| SOS1 | SOS Ras/Rac Guanine Nucleotide Exchange Factor 1 | Protein Coding | Q07889 |
| STAT3 | Signal Transducer And Activator Of Transcription 3 | Protein Coding | P40763 |
| AR | Androgen Receptor | Protein Coding | P10275 |
| KRAS | KRAS Proto-Oncogene, GTPase | Protein Coding | P01116 |
| HRAS | HRas Proto-Oncogene, GTPase | Protein Coding | P01112 |
| FAS | Fas Cell Surface Death Receptor | Protein Coding | P25445 |
| EP300 | E1A Binding Protein P300 | Protein Coding | Q09472 |
| SLC2A1 | Solute Carrier Family 2 Member 1 | Protein Coding | P11166 |
| F2 | Coagulation Factor II, Thrombin | Protein Coding | P00734 |
| NOTCH1 | Notch Receptor 1 | Protein Coding | P46531 |
| COL5A2 | Collagen Type V Alpha 2 Chain | Protein Coding | P05997 |
| TNFRSF1A | TNF Receptor Superfamily Member 1A | Protein Coding | P19438 |
| NOD2 | Nucleotide Binding Oligomerization Domain Containing 2 | Protein Coding | Q9HC29 |
| C3 | Complement C3 | Protein Coding | P01024 |
| MTHFR | Methylenetetrahydrofolate Reductase | Protein Coding | P42898 |
| AKT1 | AKT Serine/Threonine Kinase 1 | Protein Coding | P31749 |
| ADAMTS13 | ADAM Metallopeptidase With Thrombospondin Type 1 Motif 13 | Protein Coding | Q76LX8 |
| FMR1 | Fragile X Messenger Ribonucleoprotein 1 | Protein Coding | Q06787 |
| THBD | Thrombomodulin | Protein Coding | P07204 |
| ADAMTS2 | ADAM Metallopeptidase With Thrombospondin Type 1 Motif 2 | Protein Coding | O95450 |
| INS | Insulin | Protein Coding | P01308 |
| NRAS | NRAS Proto-Oncogene, GTPase | Protein Coding | P01111 |
| TH | Tyrosine Hydroxylase | Protein Coding | P07101 |
| TNF | Tumor Necrosis Factor | Protein Coding | P01375 |
| TGFBR1 | Transforming Growth Factor Beta Receptor 1 | Protein Coding | P36897 |
| PRKCD | Protein Kinase C Delta | Protein Coding | Q05655 |
| F3 | Coagulation Factor III, Tissue Factor | Protein Coding | P13726 |
| FASLG | Fas Ligand | Protein Coding | P48023 |
| IL6 | Interleukin 6 | Protein Coding | P05231 |
| TGFB2 | Transforming Growth Factor Beta 2 | Protein Coding | P61812 |
| SERPINE1 | Serpin Family E Member 1 | Protein Coding | P05121 |
| MTOR | Mechanistic Target Of Rapamycin Kinase | Protein Coding | P42345 |
| GP1BA | Glycoprotein Ib Platelet Subunit Alpha | Protein Coding | P07359 |
| IL1B | Interleukin 1 Beta | Protein Coding | P01584 |
| ALB | Albumin | Protein Coding | P02768 |
| IL10 | Interleukin 10 | Protein Coding | P22301 |
| CRP | C-Reactive Protein | Protein Coding | P02741 |
| ANXA5 | Annexin A5 | Protein Coding | P08758 |
| MEFV | MEFV Innate Immunity Regulator, Pyrin | Protein Coding | O15553 |
| SERPINC1 | Serpin Family C Member 1 | Protein Coding | P01008 |
| BDNF | Brain Derived Neurotrophic Factor | Protein Coding | P23560 |
| ELN | Elastin | Protein Coding | P15502 |
| CFB | Complement Factor B | Protein Coding | P00751 |
| MT-TL1 | Mitochondrially Encoded TRNA-Leu (UUA/G) 1 | RNA Gene |  |
| CTLA4 | Cytotoxic T-Lymphocyte Associated Protein 4 | Protein Coding | P16410 |
| HLA-DRB1 | Major Histocompatibility Complex, Class II, DR Beta 1 | Protein Coding | P01911 |
| JAK2 | Janus Kinase 2 | Protein Coding | O60674 |
| VWF | Von Willebrand Factor | Protein Coding | P04275 |
| XIAP | X-Linked Inhibitor Of Apoptosis | Protein Coding | P98170 |
| ACE | Angiotensin I Converting Enzyme | Protein Coding | P12821 |
| C1R | Complement C1r | Protein Coding | P00736 |
| CP | Ceruloplasmin | Protein Coding | P00450 |
| VEGFA | Vascular Endothelial Growth Factor A | Protein Coding | P15692 |
| IFNG | Interferon Gamma | Protein Coding | P01579 |
| MAPT | Microtubule Associated Protein Tau | Protein Coding | P10636 |
| CXCR4 | C-X-C Motif Chemokine Receptor 4 | Protein Coding | P61073 |
| ADIPOQ | Adiponectin, C1Q And Collagen Domain Containing | Protein Coding | Q15848 |
| IL1RN | Interleukin 1 Receptor Antagonist | Protein Coding | P18510 |
| MAPK1 | Mitogen-Activated Protein Kinase 1 | Protein Coding | P28482 |
| IGF1 | Insulin Like Growth Factor 1 | Protein Coding | P05019 |
| SELP | Selectin P | Protein Coding | P16109 |
| TLR4 | Toll Like Receptor 4 | Protein Coding | O00206 |
| TGFB1 | Transforming Growth Factor Beta 1 | Protein Coding | P01137 |
| EDN1 | Endothelin 1 | Protein Coding | P05305 |
| BDNF-AS | BDNF Antisense RNA | RNA Gene |  |
| CXCL8 | C-X-C Motif Chemokine Ligand 8 | Protein Coding | P10145 |
| CBS | Cystathionine Beta-Synthase | Protein Coding | P35520 |
| NR3C1 | Nuclear Receptor Subfamily 3 Group C Member 1 | Protein Coding | P04150 |
| LEP | Leptin | Protein Coding | P41159 |
| PLAT | Plasminogen Activator, Tissue Type | Protein Coding | P00750 |
| CASP8 | Caspase 8 | Protein Coding | Q14790 |
| PPARG | Peroxisome Proliferator Activated Receptor Gamma | Protein Coding | P37231 |
| SFTA3 | Surfactant Associated 3 | RNA Gene | P0C7M3 |
| PON1 | Paraoxonase 1 | Protein Coding | P27169 |
| PRL | Prolactin | Protein Coding | P01236 |
| HLA-DQB1 | Major Histocompatibility Complex, Class II, DQ Beta 1 | Protein Coding | P01920 |
| SELE | Selectin E | Protein Coding | P16581 |
| MMP2 | Matrix Metallopeptidase 2 | Protein Coding | P08253 |
| ENG | Endoglin | Protein Coding | P17813 |
| C1S | Complement C1s | Protein Coding | P09871 |
| ATP1A3 | ATPase Na+/K+ Transporting Subunit Alpha 3 | Protein Coding | P13637 |
| GBA1 | Glucosylceramidase Beta 1 | Protein Coding | P04062 |
| RBP4 | Retinol Binding Protein 4 | Protein Coding | P02753 |
| CGA | Glycoprotein Hormones, Alpha Polypeptide | Protein Coding | P01215 |
| CD40 | CD40 Molecule | Protein Coding | P25942 |
| F10 | Coagulation Factor X | Protein Coding | P00742 |
| TNFAIP3 | TNF Alpha Induced Protein 3 | Protein Coding | P21580 |
| CCL2 | C-C Motif Chemokine Ligand 2 | Protein Coding | P13500 |
| C4A | Complement C4A (Chido/Rodgers Blood Group) | Protein Coding | P0C0L4 |
| LINC02605 | Long Intergenic Non-Protein Coding RNA 2605 | RNA Gene |  |
| FLT1 | Fms Related Receptor Tyrosine Kinase 1 | Protein Coding | P17948 |
| MIR21 | MicroRNA 21 | RNA Gene |  |
| NOS3 | Nitric Oxide Synthase 3 | Protein Coding | P29474 |
| ELANE | Elastase, Neutrophil Expressed | Protein Coding | P08246 |
| SHBG | Sex Hormone Binding Globulin | Protein Coding | P04278 |
| ABCB4 | ATP Binding Cassette Subfamily B Member 4 | Protein Coding | P21439 |
| MMP9 | Matrix Metallopeptidase 9 | Protein Coding | P14780 |
| VCAM1 | Vascular Cell Adhesion Molecule 1 | Protein Coding | P19320 |
| SEPT5-GP1BB | SEPT5-GP1BB Readthrough | RNA Gene |  |
| F12 | Coagulation Factor XII | Protein Coding | P00748 |
| PRF1 | Perforin 1 | Protein Coding | P14222 |
| APOA1 | Apolipoprotein A1 | Protein Coding | P02647 |
| CSF3 | Colony Stimulating Factor 3 | Protein Coding | P09919 |
| ANXA2 | Annexin A2 | Protein Coding | P07355 |
| GNRH1 | Gonadotropin Releasing Hormone 1 | Protein Coding | P01148 |
| XIST | X Inactive Specific Transcript | RNA Gene |  |
| MIR155 | MicroRNA 155 | RNA Gene |  |
| AFP | Alpha Fetoprotein | Protein Coding | P02771 |
| NFKBIA | NFKB Inhibitor Alpha | Protein Coding | P25963 |
| B2M | Beta-2-Microglobulin | Protein Coding | P61769 |
| SEMA3A | Semaphorin 3A | Protein Coding | Q14563 |
| SOCS1 | Suppressor Of Cytokine Signaling 1 | Protein Coding | O15524 |
| GPT | Glutamic--Pyruvic Transaminase | Protein Coding | P24298 |
| IL6R | Interleukin 6 Receptor | Protein Coding | P08887 |
| FN1 | Fibronectin 1 | Protein Coding | P02751 |
| IL4 | Interleukin 4 | Protein Coding | P05112 |
| HIF1A | Hypoxia Inducible Factor 1 Subunit Alpha | Protein Coding | Q16665 |
| IL17A | Interleukin 17A | Protein Coding | Q16552 |
| PAPPA | Pappalysin 1 | Protein Coding | Q13219 |
| F8 | Coagulation Factor VIII | Protein Coding | P00451 |
| CALR | Calreticulin | Protein Coding | P27797 |
| PLA2G6 | Phospholipase A2 Group VI | Protein Coding | O60733 |
| IL2 | Interleukin 2 | Protein Coding | P60568 |
| CYP17A1 | Cytochrome P450 Family 17 Subfamily A Member 1 | Protein Coding | P05093 |
| MIRLET7C | MicroRNA Let-7c | RNA Gene |  |
| TLR2 | Toll Like Receptor 2 | Protein Coding | O60603 |
| IL1A | Interleukin 1 Alpha | Protein Coding | P01583 |
| PGF | Placental Growth Factor | Protein Coding | P49763 |
| MBL2 | Mannose Binding Lectin 2 | Protein Coding | P11226 |
| NFKB1 | Nuclear Factor Kappa B Subunit 1 | Protein Coding | P19838 |
| IGFBP1 | Insulin Like Growth Factor Binding Protein 1 | Protein Coding | P08833 |
| PWAR1 | Prader Willi/Angelman Region RNA 1 | RNA Gene |  |
| APOB | Apolipoprotein B | Protein Coding | P04114 |
| ITGB3 | Integrin Subunit Beta 3 | Protein Coding | P05106 |
| PTPN22 | Protein Tyrosine Phosphatase Non-Receptor Type 22 | Protein Coding | Q9Y2R2 |
| ICAM1 | Intercellular Adhesion Molecule 1 | Protein Coding | P05362 |
| IL18 | Interleukin 18 | Protein Coding | Q14116 |
| PF4 | Platelet Factor 4 | Protein Coding | P02776 |
| DRD2 | Dopamine Receptor D2 | Protein Coding | P14416 |
| IRF5 | Interferon Regulatory Factor 5 | Protein Coding | Q13568 |
| CRKL | CRK Like Proto-Oncogene, Adaptor Protein | Protein Coding | P46109 |
| FCGR2A | Fc Gamma Receptor IIa | Protein Coding | P12318 |
| DPYSL5 | Dihydropyrimidinase Like 5 | Protein Coding | Q9BPU6 |
| MPO | Myeloperoxidase | Protein Coding | P05164 |
| SOD1 | Superoxide Dismutase 1 | Protein Coding | P00441 |
| MAPK14 | Mitogen-Activated Protein Kinase 14 | Protein Coding | Q16539 |
| MALAT1 | Metastasis Associated Lung Adenocarcinoma Transcript 1 | RNA Gene |  |
| IKZF1 | IKAROS Family Zinc Finger 1 | Protein Coding | Q13422 |
| MAP3K7 | Mitogen-Activated Protein Kinase Kinase Kinase 7 | Protein Coding | O43318 |
| LDLR | Low Density Lipoprotein Receptor | Protein Coding | P01130 |
| CERNA3 | Competing Endogenous LncRNA 3 For MiR-645 | RNA Gene |  |
| DNASE1 | Deoxyribonuclease 1 | Protein Coding | P24855 |
| TFPI | Tissue Factor Pathway Inhibitor | Protein Coding | P10646 |
| IL2RA | Interleukin 2 Receptor Subunit Alpha | Protein Coding | P01589 |
| GAS5 | Growth Arrest Specific 5 | RNA Gene |  |
| PROCR | Protein C Receptor | Protein Coding | Q9UNN8 |
| F2RL1 | F2R Like Trypsin Receptor 1 | Protein Coding | P55085 |
| CSF2 | Colony Stimulating Factor 2 | Protein Coding | P04141 |
| MYD88 | MYD88 Innate Immune Signal Transduction Adaptor | Protein Coding | Q99836 |
| P4HB | Prolyl 4-Hydroxylase Subunit Beta | Protein Coding | P07237 |
| CCL5 | C-C Motif Chemokine Ligand 5 | Protein Coding | P13501 |
| ITGB2 | Integrin Subunit Beta 2 | Protein Coding | P05107 |
| ANGPT2 | Angiopoietin 2 | Protein Coding | O15123 |
| AGTR1 | Angiotensin II Receptor Type 1 | Protein Coding | P30556 |
| TNFRSF1B | TNF Receptor Superfamily Member 1B | Protein Coding | P20333 |
| TF | Transferrin | Protein Coding | P02787 |
| LOC106627981 | GBA Recombination Region | Functional Element | |
| F11 | Coagulation Factor XI | Protein Coding | P03951 |
| PDCD1 | Programmed Cell Death 1 | Protein Coding | Q15116 |
| TSHR | Thyroid Stimulating Hormone Receptor | Protein Coding | P16473 |
| NEAT1 | Nuclear Paraspeckle Assembly Transcript 1 | RNA Gene |  |
| NGF | Nerve Growth Factor | Protein Coding | P01138 |
| CD36 | CD36 Molecule | Protein Coding | P16671 |
| SPP1 | Secreted Phosphoprotein 1 | Protein Coding | P10451 |
| FGF2 | Fibroblast Growth Factor 2 | Protein Coding | P09038 |
| ABCA1 | ATP Binding Cassette Subfamily A Member 1 | Protein Coding | O95477 |
| BCL2 | BCL2 Apoptosis Regulator | Protein Coding | P10415 |
| IL1R1 | Interleukin 1 Receptor Type 1 | Protein Coding | P14778 |
| HMGB1 | High Mobility Group Box 1 | Protein Coding | P09429 |
| CD55 | CD55 Molecule (Cromer Blood Group) | Protein Coding | P08174 |
| CGB5 | Chorionic Gonadotropin Subunit Beta 5 | Protein Coding | P0DN86 |
| IL13 | Interleukin 13 | Protein Coding | P35225 |
| PGR-AS1 | PGR Antisense RNA 1 | RNA Gene |  |
| CXCL12 | C-X-C Motif Chemokine Ligand 12 | Protein Coding | P48061 |
| CD28 | CD28 Molecule | Protein Coding | P10747 |
| EGF | Epidermal Growth Factor | Protein Coding | P01133 |
| CCL3 | C-C Motif Chemokine Ligand 3 | Protein Coding | P10147 |
| CASP3 | Caspase 3 | Protein Coding | P42574 |
| LOC126862264 | CDK7 Strongly-Dependent Group 2 Enhancer GRCh37_chr16:3293322-3294521 | Functional Element | |
| NOS2 | Nitric Oxide Synthase 2 | Protein Coding | P35228 |
| RIPK1 | Receptor Interacting Serine/Threonine Kinase 1 | Protein Coding | Q13546 |
| FGA | Fibrinogen Alpha Chain | Protein Coding | P02671 |
| TG | Thyroglobulin | Protein Coding | P01266 |
| IFNGR1 | Interferon Gamma Receptor 1 | Protein Coding | P15260 |
| KDR | Kinase Insert Domain Receptor | Protein Coding | P35968 |
| IL5 | Interleukin 5 | Protein Coding | P05113 |
| TFRC | Transferrin Receptor | Protein Coding | P02786 |
| TEK | TEK Receptor Tyrosine Kinase | Protein Coding | Q02763 |
| CD63 | CD63 Molecule | Protein Coding | P08962 |
| MIR146B | MicroRNA 146b | RNA Gene |  |
| CCR5 | C-C Motif Chemokine Receptor 5 | Protein Coding | P51681 |
| CASP1 | Caspase 1 | Protein Coding | P29466 |
| MIR483 | MicroRNA 483 | RNA Gene |  |
| MIR221 | MicroRNA 221 | RNA Gene |  |
| HSPD1 | Heat Shock Protein Family D (Hsp60) Member 1 | Protein Coding | P10809 |
| PTGS2 | Prostaglandin-Endoperoxide Synthase 2 | Protein Coding | P35354 |
| IL3 | Interleukin 3 | Protein Coding | P08700 |
| HMOX1 | Heme Oxygenase 1 | Protein Coding | P09601 |
| TLR3 | Toll Like Receptor 3 | Protein Coding | O15455 |
| HLA-G | Major Histocompatibility Complex, Class I, G | Protein Coding | P17693 |
| ITGA2B | Integrin Subunit Alpha 2b | Protein Coding | P08514 |
| CST3 | Cystatin C | Protein Coding | P01034 |
| CPB2 | Carboxypeptidase B2 | Protein Coding | Q96IY4 |
| TLR7 | Toll Like Receptor 7 | Protein Coding | Q9NYK1 |
| TPO | Thyroid Peroxidase | Protein Coding | P07202 |
| MMP3 | Matrix Metallopeptidase 3 | Protein Coding | P08254 |
| JUN | Jun Proto-Oncogene, AP-1 Transcription Factor Subunit | Protein Coding | P05412 |
| SIRT1 | Sirtuin 1 | Protein Coding | Q96EB6 |
| ITGAM | Integrin Subunit Alpha M | Protein Coding | P11215 |
| IL1RAPL2 | Interleukin 1 Receptor Accessory Protein Like 2 | Protein Coding | Q9NP60 |
| F9 | Coagulation Factor IX | Protein Coding | P00740 |
| GSTM1 | Glutathione S-Transferase Mu 1 | Protein Coding | P09488 |
| TRIP11 | Thyroid Hormone Receptor Interactor 11 | Protein Coding | Q15643 |
| MIF | Macrophage Migration Inhibitory Factor | Protein Coding | P14174 |
| TNFSF13B | TNF Superfamily Member 13b | Protein Coding | Q9Y275 |
| CXCL10 | C-X-C Motif Chemokine Ligand 10 | Protein Coding | P02778 |
| MIR210 | MicroRNA 210 | RNA Gene |  |
| MIR145 | MicroRNA 145 | RNA Gene |  |
| MIR23A | MicroRNA 23a | RNA Gene |  |
| LTA | Lymphotoxin Alpha | Protein Coding | P01374 |
| BAX | BCL2 Associated X, Apoptosis Regulator | Protein Coding | Q07812 |
| PTPRC | Protein Tyrosine Phosphatase Receptor Type C | Protein Coding | P08575 |
| TUG1 | Taurine Up-Regulated 1 | Protein Coding | A0A6I8PU40 |
| CREB1 | CAMP Responsive Element Binding Protein 1 | Protein Coding | P16220 |
| MMP1 | Matrix Metallopeptidase 1 | Protein Coding | P03956 |
| HAMP | Hepcidin Antimicrobial Peptide | Protein Coding | P81172 |
| IL7 | Interleukin 7 | Protein Coding | P13232 |
| PCNA | Proliferating Cell Nuclear Antigen | Protein Coding | P12004 |
| DNASE1L3 | Deoxyribonuclease 1 Like 3 | Protein Coding | Q13609 |
| CLU | Clusterin | Protein Coding | P10909 |
| MIR30A | MicroRNA 30a | RNA Gene |  |
| ITGA2 | Integrin Subunit Alpha 2 | Protein Coding | P17301 |
| LIF | LIF Interleukin 6 Family Cytokine | Protein Coding | P15018 |
| MIR133B | MicroRNA 133b | RNA Gene |  |
| SOD2-OT1 | SOD2 Overlapping Transcript 1 | RNA Gene |  |
| VIM | Vimentin | Protein Coding | P08670 |
| TNFRSF11B | TNF Receptor Superfamily Member 11b | Protein Coding | O00300 |
| AGER | Advanced Glycosylation End-Product Specific Receptor | Protein Coding | Q15109 |
| MIR320A | MicroRNA 320a | RNA Gene |  |
| THBS1 | Thrombospondin 1 | Protein Coding | P07996 |
| IRF1 | Interferon Regulatory Factor 1 | Protein Coding | P10914 |
| HGF | Hepatocyte Growth Factor | Protein Coding | P14210 |
| GCH1 | GTP Cyclohydrolase 1 | Protein Coding | P30793 |
| SELL | Selectin L | Protein Coding | P14151 |
| PROZ | Protein Z, Vitamin K Dependent Plasma Glycoprotein | Protein Coding | P22891 |
| PDE2A | Phosphodiesterase 2A | Protein Coding | O00408 |
| NFE2L2 | NFE2 Like BZIP Transcription Factor 2 | Protein Coding | Q16236 |
| CAT | Catalase | Protein Coding | P04040 |
| CD14 | CD14 Molecule | Protein Coding | P08571 |
| IL2RB | Interleukin 2 Receptor Subunit Beta | Protein Coding | P14784 |
| LTF | Lactotransferrin | Protein Coding | P02788 |
| TAC1 | Tachykinin Precursor 1 | Protein Coding | P20366 |
| GSTT1 | Glutathione S-Transferase Theta 1 | Protein Coding | P30711 |
| RELA | RELA Proto-Oncogene, NF-KB Subunit | Protein Coding | Q04206 |
| F2R | Coagulation Factor II Thrombin Receptor | Protein Coding | P25116 |
| PARK7 | Parkinsonism Associated Deglycase | Protein Coding | Q99497 |
| PIK3CG | Phosphatidylinositol-4,5-Bisphosphate 3-Kinase Catalytic Subunit Gamma | Protein Coding | P48736 |
| MIR15B | MicroRNA 15b | RNA Gene |  |
| DBH | Dopamine Beta-Hydroxylase | Protein Coding | P09172 |
| TNFSF10 | TNF Superfamily Member 10 | Protein Coding | P50591 |
| MIR200C | MicroRNA 200c | RNA Gene |  |
| TRAF6 | TNF Receptor Associated Factor 6 | Protein Coding | Q9Y4K3 |
| PIEZO1 | Piezo Type Mechanosensitive Ion Channel Component 1 (Er Blood Group) | Protein Coding | Q92508 |
| GDF15 | Growth Differentiation Factor 15 | Protein Coding | Q99988 |
| LCN2 | Lipocalin 2 | Protein Coding | P80188 |
| CSF1 | Colony Stimulating Factor 1 | Protein Coding | P09603 |
| TLR9 | Toll Like Receptor 9 | Protein Coding | Q9NR96 |
| IL12RB1 | Interleukin 12 Receptor Subunit Beta 1 | Protein Coding | P42701 |
| S100A9 | S100 Calcium Binding Protein A9 | Protein Coding | P06702 |
| F13A1 | Coagulation Factor XIII A Chain | Protein Coding | P00488 |
| FGB | Fibrinogen Beta Chain | Protein Coding | P02675 |
| PLA2G7 | Phospholipase A2 Group VII | Protein Coding | Q13093 |
| MIR185 | MicroRNA 185 | RNA Gene |  |
| ADAMTSL1 | ADAMTS Like 1 | Protein Coding | Q8N6G6 |
| XDH | Xanthine Dehydrogenase | Protein Coding | P47989 |
| IL15 | Interleukin 15 | Protein Coding | P40933 |
| PTX3 | Pentraxin 3 | Protein Coding | P26022 |
| MIR326 | MicroRNA 326 | RNA Gene |  |
| VTN | Vitronectin | Protein Coding | P04004 |
| TLR6 | Toll Like Receptor 6 | Protein Coding | Q9Y2C9 |
| CCL4 | C-C Motif Chemokine Ligand 4 | Protein Coding | P13236 |
| F7 | Coagulation Factor VII | Protein Coding | P08709 |
| ADAM17 | ADAM Metallopeptidase Domain 17 | Protein Coding | P78536 |
| MAPK8 | Mitogen-Activated Protein Kinase 8 | Protein Coding | P45983 |
| MIR9-1 | MicroRNA 9-1 | RNA Gene |  |
| S100A8 | S100 Calcium Binding Protein A8 | Protein Coding | P05109 |
| LPA | Lipoprotein(A) | Protein Coding | P08519 |
| CCR2 | C-C Motif Chemokine Receptor 2 | Protein Coding | P41597 |
| CXCL1 | C-X-C Motif Chemokine Ligand 1 | Protein Coding | P09341 |
| FCGR3A | Fc Gamma Receptor IIIa | Protein Coding | P08637 |
| CDH5 | Cadherin 5 | Protein Coding | P33151 |
| KNG1 | Kininogen 1 | Protein Coding | P01042 |
| CXCR3 | C-X-C Motif Chemokine Receptor 3 | Protein Coding | P49682 |
| CD274 | CD274 Molecule | Protein Coding | Q9NZQ7 |
| IL10RA | Interleukin 10 Receptor Subunit Alpha | Protein Coding | Q13651 |
| MIR195 | MicroRNA 195 | RNA Gene |  |
| MSN | Moesin | Protein Coding | P26038 |
| CYP3A5 | Cytochrome P450 Family 3 Subfamily A Member 5 | Protein Coding | P20815 |
| CR1 | Complement C3b/C4b Receptor 1 (Knops Blood Group) | Protein Coding | P17927 |
| LAMP1 | Lysosomal Associated Membrane Protein 1 | Protein Coding | P11279 |
| HSP90AA1 | Heat Shock Protein 90 Alpha Family Class A Member 1 | Protein Coding | P07900 |
| PRKCA | Protein Kinase C Alpha | Protein Coding | P17252 |
| MIR423 | MicroRNA 423 | RNA Gene |  |
| HBEGF | Heparin Binding EGF Like Growth Factor | Protein Coding | Q99075 |
| CYBA | Cytochrome B-245 Alpha Chain | Protein Coding | P13498 |
| PLA2R1 | Phospholipase A2 Receptor 1 | Protein Coding | Q13018 |
| FXN | Frataxin | Protein Coding | Q16595 |
| SOCS3 | Suppressor Of Cytokine Signaling 3 | Protein Coding | O14543 |
| MIR98 | MicroRNA 98 | RNA Gene |  |
| PVALB | Parvalbumin | Protein Coding | P20472 |
| ANGPT1 | Angiopoietin 1 | Protein Coding | Q15389 |
| FCGR3B | Fc Gamma Receptor IIIb | Protein Coding | O75015 |
| TNXA | Tenascin XA (Pseudogene) | Pseudogene | Q16473 |
| AQP4 | Aquaporin 4 | Protein Coding | P55087 |
| ICOS | Inducible T Cell Costimulator | Protein Coding | Q9Y6W8 |
| CTSA | Cathepsin A | Protein Coding | P10619 |
| IL33 | Interleukin 33 | Protein Coding | O95760 |
| FCN2 | Ficolin 2 | Protein Coding | Q15485 |
| PLA2G2A | Phospholipase A2 Group IIA | Protein Coding | P14555 |
| CD44 | CD44 Molecule (Indian Blood Group) | Protein Coding | P16070 |
| TLR8 | Toll Like Receptor 8 | Protein Coding | Q9NR97 |
| LGALS3 | Galectin 3 | Protein Coding | P17931 |
| GP6 | Glycoprotein VI Platelet | Protein Coding | Q9HCN6 |
| ANXA1 | Annexin A1 | Protein Coding | P04083 |
| CXCL9 | C-X-C Motif Chemokine Ligand 9 | Protein Coding | Q07325 |
| IFNL1 | Interferon Lambda 1 | Protein Coding | Q8IU54 |
| PLA2G4A | Phospholipase A2 Group IVA | Protein Coding | P47712 |
| CTSD | Cathepsin D | Protein Coding | P07339 |
| IL22 | Interleukin 22 | Protein Coding | Q9GZX6 |
| CD38 | CD38 Molecule | Protein Coding | P28907 |
| MIR494 | MicroRNA 494 | RNA Gene |  |
| C1QA | Complement C1q A Chain | Protein Coding | P02745 |
| IGF2R | Insulin Like Growth Factor 2 Receptor | Protein Coding | P11717 |
| HLA-E | Major Histocompatibility Complex, Class I, E | Protein Coding | P13747 |
| CXCL13 | C-X-C Motif Chemokine Ligand 13 | Protein Coding | O43927 |
| TREM1 | Triggering Receptor Expressed On Myeloid Cells 1 | Protein Coding | Q9NP99 |
| ADM | Adrenomedullin | Protein Coding | P35318 |
| TRA-TGC7-1 | TRNA-Ala (Anticodon TGC) 7-1 | RNA Gene |  |
| FOXO1 | Forkhead Box O1 | Protein Coding | Q12778 |
| IDO1 | Indoleamine 2,3-Dioxygenase 1 | Protein Coding | P14902 |
| APLN | Apelin | Protein Coding | Q9ULZ1 |
| CHI3L1 | Chitinase 3 Like 1 | Protein Coding | P36222 |
| CEBPB | CCAAT Enhancer Binding Protein Beta | Protein Coding | P17676 |
| SRSF1 | Serine And Arginine Rich Splicing Factor 1 | Protein Coding | Q07955 |
| CCR6 | C-C Motif Chemokine Receptor 6 | Protein Coding | P51684 |
| PROC | Protein C, Inactivator Of Coagulation Factors Va And VIIIa | Protein Coding | P04070 |
| MIR381 | MicroRNA 381 | RNA Gene |  |
| SDC1 | Syndecan 1 | Protein Coding | P18827 |
| CCR7 | C-C Motif Chemokine Receptor 7 | Protein Coding | P32248 |
| PC | Pyruvate Carboxylase | Protein Coding | P11498 |
| ERVW-1 | Endogenous Retrovirus Group W Member 1, Envelope | Protein Coding | Q9UQF0 |
| PROS1 | Protein S | Protein Coding | P07225 |
| MIR346 | MicroRNA 346 | RNA Gene |  |
| MIR518B | MicroRNA 518b | RNA Gene |  |
| KRT7 | Keratin 7 | Protein Coding | P08729 |
| NRP1 | Neuropilin 1 | Protein Coding | O14786 |
| HAVCR2 | Hepatitis A Virus Cellular Receptor 2 | Protein Coding | Q8TDQ0 |
| CALB1 | Calbindin 1 | Protein Coding | P05937 |
| CD69 | CD69 Molecule | Protein Coding | Q07108 |
| TLR1 | Toll Like Receptor 1 | Protein Coding | Q15399 |
| KLF4 | KLF Transcription Factor 4 | Protein Coding | O43474 |
| CCR4 | C-C Motif Chemokine Receptor 4 | Protein Coding | P51679 |
| FGR | FGR Proto-Oncogene, Src Family Tyrosine Kinase | Protein Coding | P09769 |
| CYP2E1 | Cytochrome P450 Family 2 Subfamily E Member 1 | Protein Coding | P05181 |
| ITGA5 | Integrin Subunit Alpha 5 | Protein Coding | P08648 |
| TLR5 | Toll Like Receptor 5 | Protein Coding | O60602 |
| FOXO3 | Forkhead Box O3 | Protein Coding | O43524 |
| ITGA1 | Integrin Subunit Alpha 1 | Protein Coding | P56199 |
| ATF2 | Activating Transcription Factor 2 | Protein Coding | P15336 |
| CREM | CAMP Responsive Element Modulator | Protein Coding | Q03060 |
| IL9 | Interleukin 9 | Protein Coding | P15248 |
| SERPINB2 | Serpin Family B Member 2 | Protein Coding | P05120 |
| SOCS2 | Suppressor Of Cytokine Signaling 2 | Protein Coding | O14508 |
| MIR517A | MicroRNA 517a | RNA Gene |  |
| C1QBP | Complement C1q Binding Protein | Protein Coding | Q07021 |
| FCGR1A | Fc Gamma Receptor Ia | Protein Coding | P12314 |
| SERPINA10 | Serpin Family A Member 10 | Protein Coding | Q9UK55 |
| GAB2 | GRB2 Associated Binding Protein 2 | Protein Coding | Q9UQC2 |
| PDYN | Prodynorphin | Protein Coding | P01213 |
| MIR654 | MicroRNA 654 | RNA Gene |  |
| TIMP3 | TIMP Metallopeptidase Inhibitor 3 | Protein Coding | P35625 |
| TRADD | TNFRSF1A Associated Via Death Domain | Protein Coding | Q15628 |
| MIR543 | MicroRNA 543 | RNA Gene |  |
| MCAM | Melanoma Cell Adhesion Molecule | Protein Coding | P43121 |
| CISH | Cytokine Inducible SH2 Containing Protein | Protein Coding | Q9NSE2 |
| MIR433 | MicroRNA 433 | RNA Gene |  |
| ORM1 | Orosomucoid 1 | Protein Coding | P02763 |
| CD22 | CD22 Molecule | Protein Coding | P20273 |
| BID | BH3 Interacting Domain Death Agonist | Protein Coding | P55957 |
| ALCAM | Activated Leukocyte Cell Adhesion Molecule | Protein Coding | Q13740 |
| S100A10 | S100 Calcium Binding Protein A10 | Protein Coding | P60903 |
| MIR638 | MicroRNA 638 | RNA Gene |  |
| AOC3 | Amine Oxidase Copper Containing 3 | Protein Coding | Q16853 |
| ERVFRD-1 | Endogenous Retrovirus Group FRD Member 1, Envelope | Protein Coding | P60508 |
| ISG20 | Interferon Stimulated Exonuclease Gene 20 | Protein Coding | Q96AZ6 |
| FGG | Fibrinogen Gamma Chain | Protein Coding | P02679 |
| ANXA2R | Annexin A2 Receptor | Protein Coding | Q3ZCQ2 |
| MIR520A | MicroRNA 520a | RNA Gene |  |
| ST20 | Suppressor Of Tumorigenicity 20 | RNA Gene | Q9HBF5 |
| HTRA3 | HtrA Serine Peptidase 3 | Protein Coding | P83110 |
